# Cerebrospinal Fluid Histamine Levels in Healthy Children and Potential Implication for SIDS: Observational Study in a French Tertiary Care Hospital

**DOI:** 10.3389/fped.2022.819496

**Published:** 2022-04-05

**Authors:** Sabine Plancoulaine, Aurore Guyon, Clara-Odilia Inocente, Philippine Germe, Min Zhang, Philippe Robert, Jian-Sheng Lin, Patricia Franco

**Affiliations:** ^1^Université de Paris Cité, Inserm, INRAE, CRESS, Paris, France; ^2^Integrative Physiology of the Brain Arousal System, CRNL, INSERM-U1028, CNRS UMR5292, University Lyon 1, Lyon, France; ^3^Bioprojet Biotech, Saint-Grégoire, France; ^4^Pediatric Sleep Unit, Hôpital Femme Mère Enfant, Hospices Civils de Lyon, University Lyon 1, Lyon, France

**Keywords:** histamine, maturation, sleep, children, infant, SIDS

## Abstract

**Objective:**

A defect of the waking systems could constitute a factor of vulnerability for sudden infant death syndrome (SIDS). A decrease in orexin levels, which promotes wakefulness and activates histaminergic neurons (another hypothalamic wake-promoting system) has already been demonstrated between 2 and 6 months. This work aims to study the levels of histamine (HA), tele-methylhistamine (t-MeHA), its direct metabolite, and t-MeHA/HA ratio in the cerebrospinal fluid (CSF) of healthy children, to evaluate the maturation of the histaminergic system and its possible involvement in SIDS.

**Methods:**

Seventy Eight French children between 0 and 20 years (48.7% boys) were included, all of whom had a clinical indication for lumbar puncture, but subsequently found to be normal. Measurements of HA and t-MeHA in CSF were performed by reverse phase liquid chromatography coupled to mass spectrometry detection. Statistical analyses were performed using Spearman correlations and Non-parametric pairwise ranking tests.

**Results:**

A negative correlation was found between age and CSF HA (*r* = −0.44, *p* < 10^−4^) and t-MeHA (*r* = −0.70, *p* < 10^−4^) levels. In pairwise comparisons, no difference in CSF HA and t-MeHA levels was observed between youngest age groups (i.e., 0–2 mo vs. 3–6 mo), but CSF HA and t-MeHA levels were significantly lower in older children (i.e., >6 mo vs. 0–6 mo). The CSF HA decrease with age was only observed in boys, who also presented global lower CSF HA levels than girls.

**Conclusion:**

CSF HA and t-MeHA levels decrease with age in boys, and global levels are lower in boys than in girls. These results reveal changes in histaminergic transmission and metabolism during maturation. Whether lower CSF histamine values in boys compared to girls could contribute to their higher risk of SIDS warrants further research.

## Introduction

Sudden Infant Death Syndrome (SIDS) consists in the sudden and unexpected death of a child under 1 year old and usually beyond the perinatal period, which remains unexplained after an extensive investigation, including a complete autopsy and analysis of death circumstances and anterior clinical history (1). SIDS remains the main cause of post-neonatal mortality, with a rate of 38 deaths per 100,000 live births in 2016 in the United States, accounting for 42% of Sudden Unexpected Infant Deaths (SUID) (2). SIDS is a multifactorial entity (3). Main known risk factors are prenatal (born premature, maternal smoking, male) and postnatal (sleep position, infections, sleep deprivation …) during the critical period of development defined between 2 and 6 months (4, 5).

Despite intensive and long-lasting research, the underlying mechanisms involved in SIDS remain unclear. Arousability from sleep could provide a protective mechanism for survival and the temporal association between SIDS and sleep periods suggests that when confronted with a life-threatening challenge during sleep, this vital response may be impaired in infants who succumb to SIDS. Infants who subsequently died of SIDS moved less during sleep and aroused less frequently; furthermore, these observations occurred predominantly during the last part of the night, when most deaths from SIDS occur (6, 7). It has been shown that infants who subsequently became victims of SIDS not only aroused less from sleep than control infants, but had different arousal characteristics (8). Compared to a group of age-matched control infants, these SIDS victims had significantly more subcortical activations during the first part of the night between 9:00 p.m. and midnight, and fewer cortical arousals during the latter part of the night, suggesting an incomplete arousal process (8). On the other hand, all known major risk factors for SIDS such as prone sleep position, *in utero* tobacco exposure, preterm births,… have been consistently associated with decrease in both spontaneous and induced arousals from sleep (9–11).

Arousal requires a convergent action of assorted systems, such as brainstem cholinergic, aminergic neurons and some neuropeptides. Patho-anatomical studies in SIDS infants demonstrated diffuse lesions (gliosis, hypoplasia or apoptosis) within different brain structures, essentially the brainstem (12). To date, the most robust evidence has been associated with the medullary 5-hydroxytryptamine system (5-HT), with abnormalities found in ~50 to 75% of SIDS victims including decreased 5-HT1A receptor bindings and serotonin levels (13). The medullary 5-HT system is considered critical for the modulation and integration of diverse homeostatic functions such as respiratory, cardiovascular and arousal controls.

Orexin/hypocretin and histamine (HA) neurons, located proximately in the posterior hypothalamus, constitute two hypothalamic wake-promoting systems (14). Under physiological conditions, they must cease their activity to allow the initiation and maintenance of sleep, probably thanks to multiple GABA inhibitory inputs (15). With reference to SIDS, an impairment of either system could lead to a defect of arousal. The neuropeptides orexins contribute to behavioral aspects of wakefulness, including maintenance of muscle tone and posture, locomotion, food intake and emotional responses (14, 16). Orexins therefore promotes wakefulness through locomotion and behavioral activation. We recently found that the period of major incidence of SIDS (2–6 months) matches with a decrease in CSF orexin levels (17) which could trigger a more important vulnerability facing SIDS with difficulties to awaken.

Orexin neurons project directly to the tuberomammillary nucleus and depolarize HA neurons, another hypothalamic widespread projecting and wake-promoting system whose deficiency is associated with sleepiness in animal and human models of sleep disorders such as narcolepsy (18–20). Concerning SIDS, a decrease in orexin excitatory inputs could entail a parallel decline in HA levels between 2 and 6 months.

In the mammalian brain system, HA is exclusively synthesized by histidine decarboxylase before being inactivated after release by histamine N-methyltransferase into its sole direct metabolite: tele-methylhistamine (t-MeHA). The latter is stable during several hours in the CSF, hence HA, t-MeHA and the ratio between t-MeHA/HA constitute a mirror of the brain HA turnover and transmission (21, 22). Although HA levels in adult sleep disorders such as narcolepsy remains controversial (22–26), we recently showed in childhood narcolepsy at the early stage of the decease has shown an impaired HA turnover and neurotransmission. These results together with those in animal studies indicate that HA could be involved in various sleep disorders with impaired vigilance.

The objective of the present study was therefore to better understand the maturation of the cerebral histaminergic system during childhood, *via* measurement of HA, t-MeHA levels and t-MeHA/HA ratio in patients between 0 and 20 years old with focus on potential links between an impairment in maturation and SIDS. Indeed, a decline in orexin between 2 and 6 months may have an effect on HA levels at the same period by decreasing excitatory inputs.

## Materials and Methods

### Patients

The CSF samples of 91 children were collected at the Hôpital Femme Mère Enfant in Lyon, France. At the hospital admission, parents were informed that biological samples from their children could be used for research purposes and they signed a consent form.

Children born premature were excluded to avoid potential biases due to a lack of maturation of the nervous system and consequently of HA production (*N* = 6) as well as those with a documented meningitis (*N* = 7) resulting in a sample of 78 children aged 0 to 20 years old.

For children ≤ 1 year old (*n* = 42), indications for lumbar puncture were available for *N* = 30 and were fever (*N* = 24), febrile seizures (*N* = 2), acute gastroenteritis with dehydration (*N* = 2), respiratory distress by meconium. For older children (*N* = 36), medical records were available for 32 of them and indications for lumber punction were headache (*N* = 6), visual disorders (diplopia, scotoma…) (*N* = 6), epilepsy (*N* = 3), sensory-motor deficit of the lower limbs (*N* = 3), gait disturbances (*N* = 2), fever (*N* = 1), epileptic encephalopathy (*N* = 1), moya-moya disease (*N* = 1), 6-month check-up after encephalitis (*N* = 1), peripheral facial palsy (*N* = 1), arthritis (*N* = 1), acute bronchitis (*N* = 1), intrathecal infusion pump filling (*N* = 1), cranial trauma (*N* = 1), cystitis (*N* = 1), paresthesia (*N* = 1) and transitory isolated behavioral disorder (*N* = 1). All biological results were subsequently found to be normal.

### Histamine Assay

The CSF samples were immediately preserved at −80°C after sampling. Blood contamination was checked visually and specimens of CSF with abnormal color were excluded from the study. The HA and t-MeHA assays were carried out simultaneously, on CSF samples without abnormal color at visual checking, at Bioprojet-Biotech in Saint Gregoire (France) using the analytical method internally developed by Croyal et al. (25). These measurements rely on derivatization of primary amines using 4-bromobenzenesulfonyl chloride and subsequent analysis by reverse liquid chromatography (UPLC: ultra-performance liquid chromatography) with mass spectrometry detection. The combination of these two methods makes it possible to solve the problem inherent in the sensitive analysis of two amines with close physicochemical properties (26). All measurements were performed blindly. The ratio of t-MeHA/HA was calculated.

### Statistical Analysis

Children age was considered in the statistical analyses both as a continuous and a categorical variable. Age categories were then 0–2 months (*n* = 20), 2–6 months (*n* = 15), 6–12 months (*n* = 7), 1–10 years (*n* = 13), and 10–20 years (*n* = 23). The 2–6 months age group was the one of specific interest to study potential implication in SIDS.

Correlations between age and CSF measures were assessed by Spearman correlation test. Comparisons between CSF measures (HA, t-MeHA and t-MeHA/HA) according to age groups were performed using Non-parametric Kruskal-Wallis tests globally and based on pairwise rankings corrected for multiple comparisons when considering pairwise two-sided analysis.

All statistical analyses were performed using SAS 9.4. The significance level was set at *p* < 0.05.

## Results

Children were aged 1 week to 20 years and boys represented 48.7% of the population. There was no sex difference according to age groups (*p*_chisq−exact_ = 0.77). The characteristics according to age categories are presented in Table 1.

**Table 1:** Population characteristics and CSF measures by age group.

**Age group**	***N***	**Age (weeks)**	**Boys**	**CSF-HA (pM)**	**CSF-t-MeHA (pM)**	**CSF-t-MeHA/HA**
		**Median (min-max)**	**(%)**	**Median (min-max)**	**Median (min-max)**	**Median (min-max)**
≤2 months	20	4.5 (1.0–8.0)	50	706.33 (207.30–2,166.88)	5,870.15 (2,576.77–13,376.75)	7.59 (2.02–20.52)
[2–6] months	15	12.0 (9.0–26.0)	47	642.58 (183.14–3,208.27)	5,386.82 (1,293.42–10,179.70)	7.37 (0.68–35.11)
[6–12] months	7	40.0 (32.0–52.0)	71	565.17 (342.82–809.75)	3,965.26 (2,612.06–8,509.37)	7.80 (4.10–14.86)
[1–10] years	13	426.0 (246.0–483.0)	38	442.21 (27.32–1,192.80)	2,379.59 (566.55–9,648.69)	6.78 (0.78–133.06)
>10 years	23	734.0 (552.0–1,034.0)	48	297.97 (10.92–1,596.74)	1,192.31 (498.23–7,413.11)	3.81 (0.42–123.10)

*CSF, cerebrospinal fluid; HA, histamine; t-MeHA, t-methylhistamine*.

**Figure 1:**
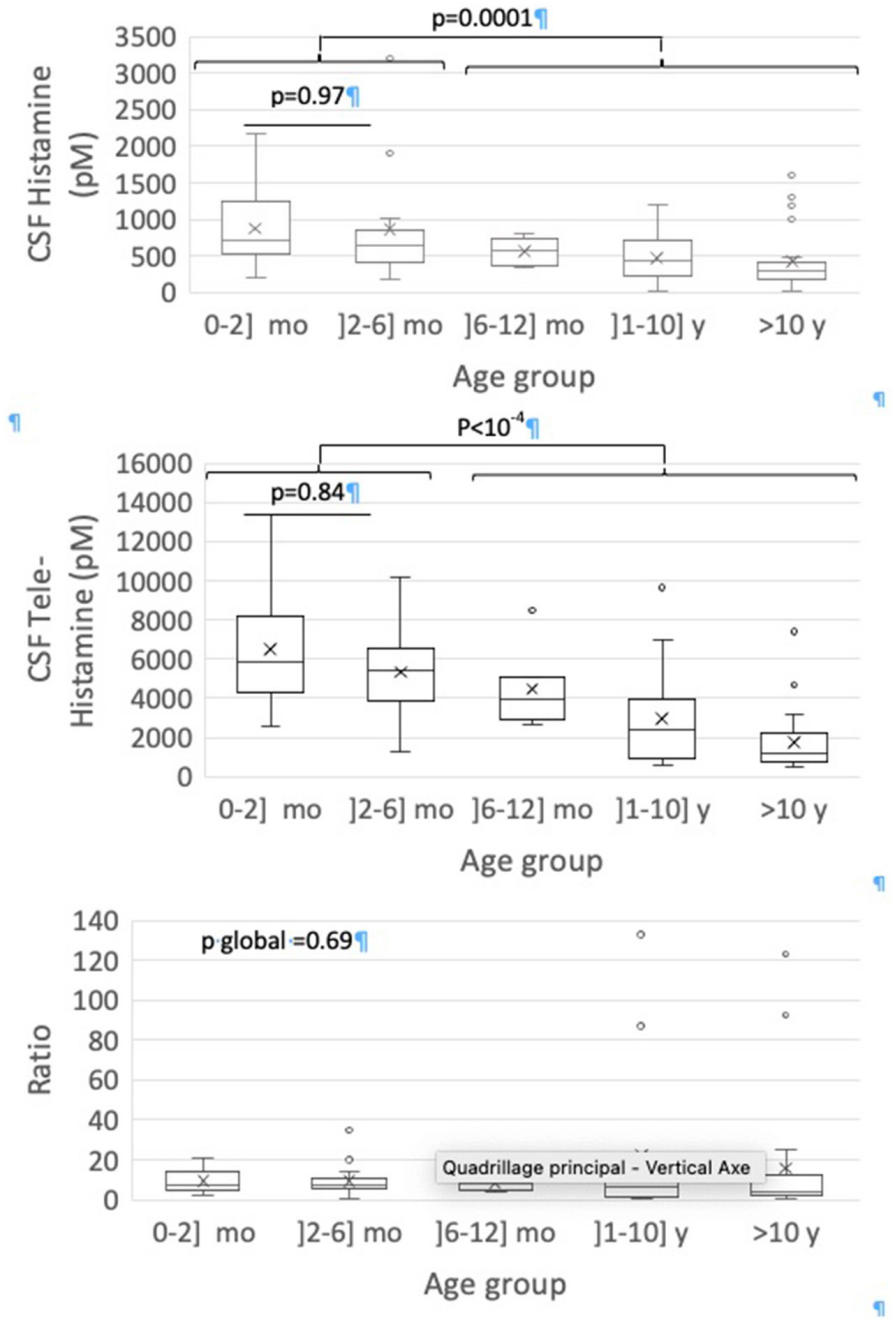
Box-and-whisker plots for CSF measures according to age groups. The minimum value, 25^th^ percentile, median, 75^th^ percentile, and the maximum value are represented. The cross is the mean value.

Global CSF HA, CSF t-MeHA levels and their ratio according to age groups are presented in Figure 1. Significant negative correlation was observed between age and CSF HA (rho = −0.44, *p* < 10^−4^) and t-MeHA levels (rho = −0.70, *p* < 10^−4^). No correlation was observed between age and t-MeHA/HA ratio (Supplementary Figure). Significant global differences for HA and t-MeHA levels were observed according to age groups (*p* = 0.001 and *p* < 10^−4^, respectively). The 2x2 group comparisons showed no significant difference for HA and t-MeHA levels between 0–2 and 2–6 months (*p* = 0.98 and *p* = 0.84, respectively) but significant decrease in both HA and t-MeHA levels between groups aged up to 6 months and the older ones (Figure 1).

**Figure 2:**
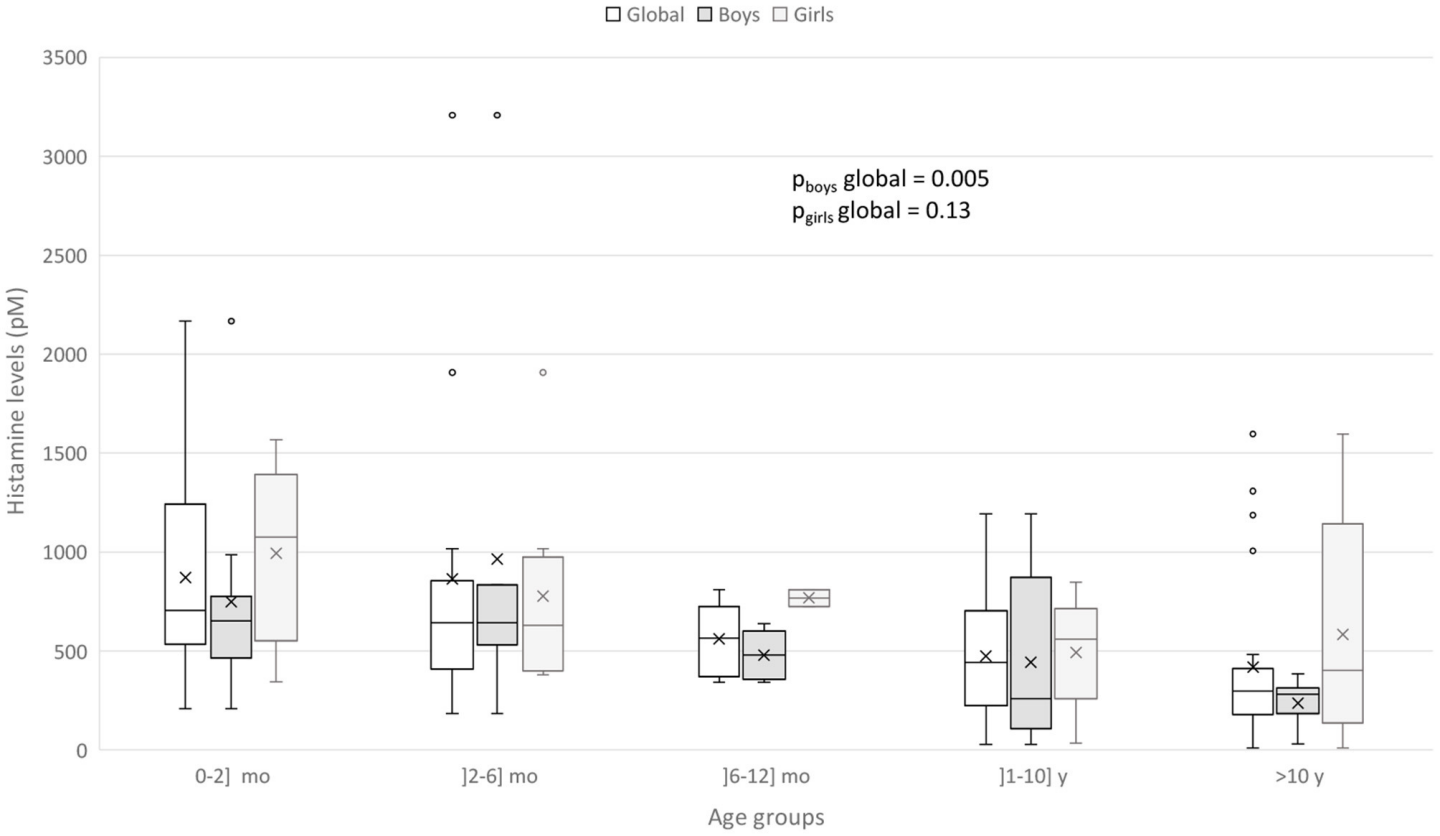
Box-and-whisker plot for CSF HA according to sex and age groups. The minimum value, 25^th^ percentile, median, 75^th^ percentile, and the maximum value are represented. The cross is the mean value.

CSF HA levels were globally lower in boys than in girls (median [25–75%] 432.57 [258.85–643.48] vs. 680.99 [383.89–1016.19], *p* = 0.03). No other difference according to sex was observed. In stratified analysis by sex, the same global relations for CSF HA levels and age groups were observed in boys (*p* = 0.005) but not in girls (*p* = 0.13) (Figure 2).

Sensitivity analyses excluding outliers (*n* = 6) provided similar results plus a significant correlation between age and t-MeHA/HA ratio when considered as continuous variables (rho = −0.31, *p* = 0.006).

## Discussion

### Main Findings

The present study provides measures of the assay of HA and its metabolite, t-MeHA and t-MeHA/HA ratio in CSF in healthy patients. The purpose is, on the one hand, to obtain normative data in order to better understand the maturation of the histaminergic system during childhood under physiological conditions, and on the other hand, to attempt to relate these results to SIDS.

We have highlighted high levels of CSF HA and t-MeHA at age before 6 months, then a progressive decrease toward the adult levels around 10 years old (*p* < 10^−4^). The difference in HA levels is more pronounced on either side of an artificial limit of 6 months old, since we do not find any statistically significant difference by comparing two-by-two lower age groups or upper age groups. Nevertheless, this decrease in HA levels after the age of 6 months should be interpreted within a general downward trend between 0 and 20 years, with the negative correlation found between age and HA levels. These results match with a previous study by Dauvilliers et al. (26), showing that patients with high HA levels are significantly younger than patients with low HA levels (*p* = 0.03) with a negative correlation between age and HA levels (*r* = −0.22, *p* < 0.005), as we found. However, that study included a wider population of patients from 4 to 86 years old. Another rare study also finds higher t-MeHA levels at 3 months old and then a subsequent progressive decrease during 15 y (27). Thus, the negative correlation that we found concerning healthy children from 0 to 20 years old seems to be maintained in adulthood. In our study, the CSF HA decrease with age is mostly driven by the CFS-HA decrease in boys, not observed in girls (Figure 2). This sex difference was not studied in the two above cited studies (26). We also detected a negative correlation between CSF t-MeHA levels and age (*p* < 10^−4^), consonant with previous findings (22). The decrease of CSF HA and t-MeHA levels and t-MeHA/HA ratio suggest possible changes in the histaminergic transmission and metabolism over the child's brain development.

### Interpretation

Several assumptions can be made about the origin of the decline of HA and t-MeHA levels. Synaptic space between neurons is known to be wider in children. HA production could therefore be more important in children to ensure an effective transmission to post-synaptic neurons. Furthermore, histamine is also produced by mast cells. An increase of the number of mast cells in children could result in a rise of the production of histamine during childhood. Changes in the choroid plexus characteristics during postnatal life could be implicated in the cerebrospinal fluid clearance (28) with a potential increased rate of entry of substances from the systemic circulation into CSF at this age (29). Such assumptions require next in-depth investigations.

Pitolisant is a potent, selective histamine 3 (H3)-receptor antagonist/inverse agonist that increases histamine synthesis, release, and transmission in the brain (30–32). Recently, we showed in a pharmacokinetic study that after a simple dose, the maximum serum concentration (Cmax) and area under the serum concentration-time curve from time zero to time of last sample collection (AUC0–10 h) were markedly higher in the younger pediatric subgroup (aged 6–<12 years) relative to older pediatric participants (aged 12–<18 years) and young adults (aged 18–45 years) suggesting in the same way a maturation in the histaminergic metabolism (33).

Regarding SIDS, the group of interest between 2 and 6 months did not have an HA level significantly different from the lower age groups (0–2 months). There is no decrease in HA during the critical time period of the peak incidence of SIDS, like we found for orexin (17), to amplify the vulnerability to SIDS between 2 and 6 months with a failure in the two arousal systems. Even so the absence of decline of HA levels in the 2–6 months group may seem unexpected at the sight of results from previous studies. Indeed, the consensus is that orexin directly excites histaminergic neurons and it has been shown that orexin levels are significantly lower at this period of child's development (17). The lack of excitatory inputs on histaminergic neurons should result in an impairment in histaminergic transmission. However, our results tally with the findings of a normal histaminergic transmission in orexin knock-out mice, similar to that of wild-type mice; as evidenced by measurement of cortical HA and t-MeHA levels (34). Thus, a decrease of orexin would not necessarily affect the histamine levels and its transmission, thanks to the likely existence of compensatory mechanisms of the brain arousal systems organized in redundant neuronal networks. We found that infants <6 months of age had higher CSF HA and t-MeHA levels than infants older than 6 months. These high levels could reflect a higher activity of the histaminergic system at this age and could constitute a factor of protection for SIDS, an idea that is supported by animal studies. Indeed, HA neurons are sensitive to high serum CO2 (hypercapnia), low pH medium and hypoxia and enhance their activity facing such situations (35, 36) that may be associated with SIDS (12). However as there were few patients between 6 and 12 months, necessary cautions should be made. As the decrease of orexin levels and the increase of HA did not take place in the exactly same period likely 2–6 months, we could suggest at this point that there is no relation between the decreased level of orexin and the increase in histamine. We unfortunately do not have joint measurements of both CSF HA and orexin in the very same patients to actually study whether the infants having a decrease in orexin levels do not have a decrease or an increase in HA levels, as we suggest here. A study of this kind would allow more affirmative interpretations. The difficulty in linking SIDS up to HA also lies on the postmortem measurements of CSF HA and t-MeHA in victim infants of SIDS. Indeed, their levels cannot be interpreted since they systematically rise significantly after death, probably due to a postmortem release from mast cells. A solution could then be the study of the number of histaminergic neurons in SIDS infants as it has already been done by Hunt et al. for orexin (37).

We found that boys presented global lower CSF HA levels than girls and that HA decrease with age was only observed in boys. From epidemiological studies, male infants have a 50% increased risk of dying of SIDS than female infants (38). This gender risk is not well understood. Dysfunction in the 5-hydroxytryptamine system, critical for the modulation and integration of diverse homeostatic functions such as respiratory, cardiovascular and arousal controls, has been implicated in the vulnerability of these future SIDS infants (13). Intrinsic factors such as prematurity, male sex, *in utero* tobacco exposure could decrease 5-hydroxytryptamine1A receptor binding density affecting the underlying vulnerability in these infants. The higher risk for males could also be explained by genetic factors (39). A modeling, using a single X-linked gene locus with a dominant allele (*p* = 1/3) for protective of cerebral anoxia, predicted the observed male excess SIDS susceptibility in US (39). On the other hand, the tachykinin neurokinin-1 receptor (NK1R) which plays a relevant role in the mediation of the chemoreceptor reflex in response to hypoxia in the medulla seem to be altered in males (40). Boys seem also to have more immature sleep than girls (41). The lower levels of HA and t-MeHA could also reflect an impairment of histamine system in male infants.

### Limitations

One of the study limitations concerns the selection of the population. Indeed, getting CSF samples of healthy patients is not an easy task whatsoever since parents as children are rather reluctant to do a lumbar puncture, this procedure being an invasive act used only to rule out or diagnose a pathology. This is why the choice was made to use CSF samples from patients admitted to the emergency department, for whom a lumbar puncture had to be performed during the care. However, to limit potential bias, only the CSF samples whose results came back normal were kept for analysis in the present study (22). In addition, the population recruitment pattern is similar to that of other studies using human CSF (17, 22). It should also be kept in mind that HA is not produced solely by the tuberous-mamillary neurons but also by the basophilic and the mast cells. Consequently, we only chose samples without the least trace of blood so as to avoid any blood contamination that could lead to erroneous data. Furthermore, we managed to exclude the role of the time of lumbar puncture as a potential confounding factor in the assay of HA and t-MeHA levels and no association was found between the time of lumbar puncture (day vs. night) and HA or t-MeHA levels. Indeed, children were awake when the CSF sample was taken. Another study limitation is the number of included children that did not allow stratified statistical analysis by sex and age-groups. However, this study sample in children is one of the largest published. Finally, in this retrospective study, we did not collect serum histamine in the population involved. However, in view of the very high serum levels of serotonin in SIDS (42) and the association of HA with hypercapnia, it would be of particular interest to accomplish a study to assess whether there are any interactions between brain and peripheral HA and serotonin.

## Conclusion

We report in the present study the CSF HA and t-MeHA levels in healthy children from 0 to 20 years old. We therefore obtained normative data in order to better understand the maturation of the histaminergic system. CSF HA levels seem to be high in infant and then to decrease during childhood with age, as t-MeHA levels do. These results suggest changes in the histaminergic transmission and metabolism over the child's brain development, that we should further investigate. These normative data can be put into perspective with measurements under pathological conditions so as to better appreciate the role and adaptations of the histaminergic system in various pathologies. Regarding SIDS, this study did not show any decrease of HA levels concurrently with that of orexin levels during the physiological period at risk for SIDS between 2 and 6 months. There is therefore no defect of the histaminergic system at this period that could have been accountable for a greater vulnerability toward SIDS. Contrary, we found that the histaminergic system seems to be highly activated during the first 6 months of age and could be a protector factor of SIDS during this high-risk period of SIDS. As the decrease of orexin levels and the increase of HA did not take place in the same period likely 2–6 months, this increase probably do not correspond to compensatory mechanisms of histaminergic neurons facing lack of orexin. Further studies with measurements of orexin and histamine levels in the CSF of the same healthy children or on evaluation of orexin and histamine neurons in the hypothalamus on SIDS victims could help to understand the physiology of these awakening systems and their potential role in the pathophysiology of SIDS.

## Data Availability Statement

The raw data supporting the conclusions of this article will be made available by the authors, without undue reservation.

## Ethics Statement

Ethic review and approval was not required for this study on human participant as samples and information on children were collected in clinical situation and for diagnosis purpose in accordance with the local legislation and institutional requirements. At the hospital admission, parents were informed that biological samples from their children could be used for research purposes and they signed a consent form.

## Author Contributions

PF and JSL contribute to the conception and design of the study and interpret the results. AG, COI, and MZ collected and managed the data. SP performed the statistical analyses and interpret the results. PG wrote the first draft of the manuscript. PR interpret the results. All authors contributed to manuscript revision, read and approved the submitted version.

## Conflict of Interest

PF received funds for speaking and board engagements with UCB pharma and Biocodex. She is co-investigator in studies promoted by Bioprojet (Pitolisant) and Jazz (Sunosi). J-SL received funds for speaking and board engagements with Bioprojet. The remaining authors declare that the research was conducted in the absence of any commercial or financial relationships that could be construed as a potential conflict of interest.

## Publisher's Note

All claims expressed in this article are solely those of the authors and do not necessarily represent those of their affiliated organizations, or those of the publisher, the editors and the reviewers. Any product that may be evaluated in this article, or claim that may be made by its manufacturer, is not guaranteed or endorsed by the publisher.
