# Supplementary material for: Cerebrospinal Fluid Histamine Levels in Healthy Children and Potential Implication for SIDS: Observational Study in a French Tertiary Care Hospital
Source: Front Pediatr. 2022 Apr 5;10:819496. doi: 10.3389/fped.2022.819496 (PMC9016218; doi:10.3389/fped.2022.819496)

Supplementary Figure 1. Scatter plot for CSF measures according to age. Spearman correlation coefficients rho =-0.44 (<10^{-4}), =-0.70 (p<10^{-4}) and =-0.20 (p=0.07) between age and CSF Histamine (HA), tele-Methylhistamine (t-MeHA) and their ratio (t-MeHA/HA) levels, respectively.

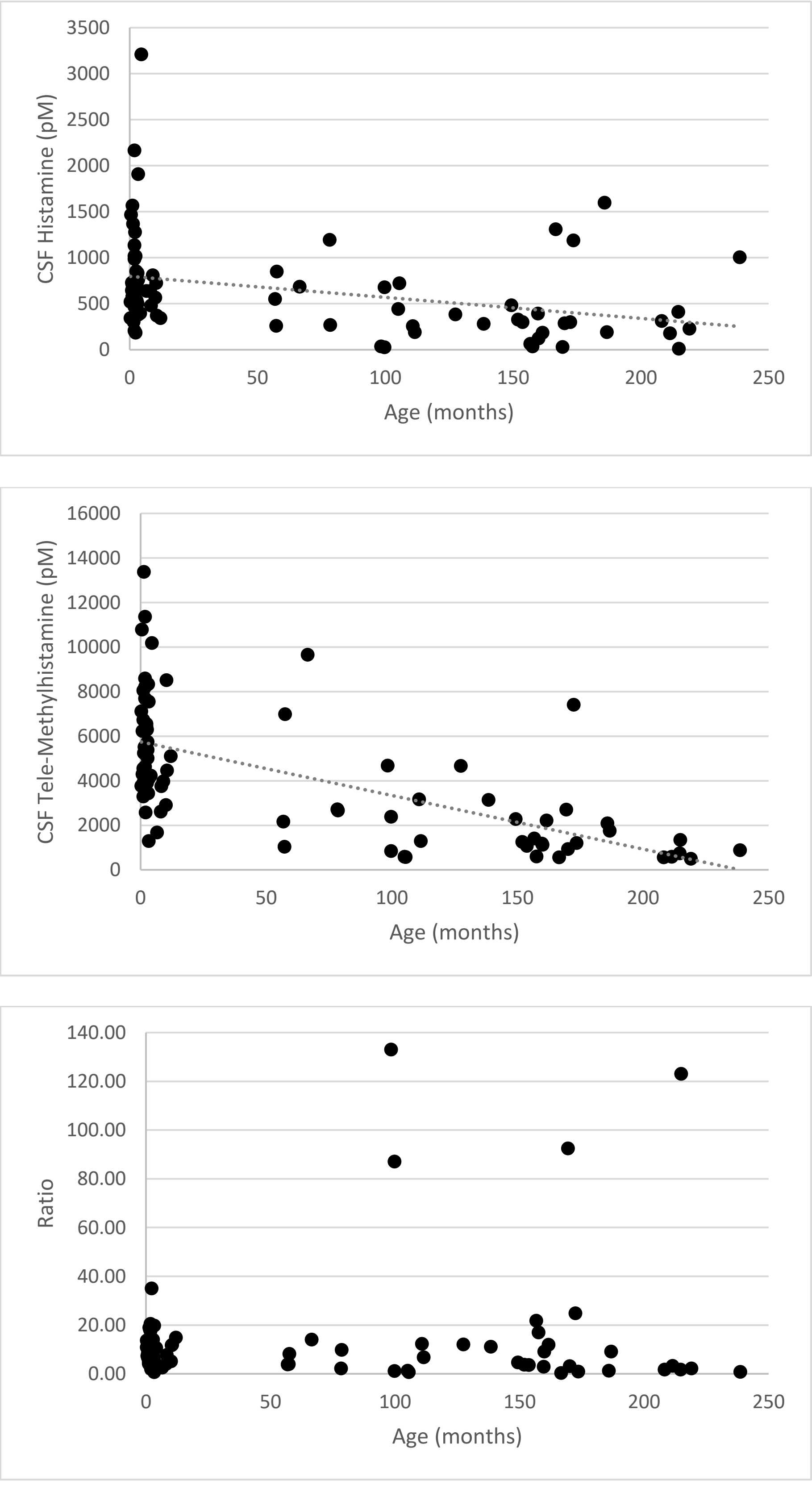

Supplement: Supplementary file 1 [file Image_1.pdf]
